# Effect of stimulated erythropoiesis on liver SMAD signaling pathway in iron-overloaded and iron-deficient mice

**DOI:** 10.1371/journal.pone.0215028

**Published:** 2019-04-08

**Authors:** Jana Frýdlová, Daniel W. Rogalsky, Jaroslav Truksa, Emanuel Nečas, Martin Vokurka, Jan Krijt

**Affiliations:** 1 Institute of Pathological Physiology, First Faculty of Medicine, Charles University, Prague, Czech Republic; 2 Laboratory of Tumour Resistance, Institute of Biotechnology, BIOCEV Research Center, Czech Academy of Sciences, Vestec, Czech Republic; Lady Davis Institute for Medical Research, CANADA

## Abstract

Expression of hepcidin, the hormone regulating iron homeostasis, is increased by iron overload and decreased by accelerated erythropoiesis or iron deficiency. The purpose of the study was to examine the effect of these stimuli, either alone or in combination, on the main signaling pathway controlling hepcidin biosynthesis in the liver, and on the expression of splenic modulators of hepcidin biosynthesis. Liver phosphorylated SMAD 1 and 5 proteins were determined by immunoblotting in male mice treated with iron dextran, kept on an iron deficient diet, or administered recombinant erythropoietin for four consecutive days. Administration of iron increased liver phosphorylated SMAD protein content and hepcidin mRNA content; subsequent administration of erythropoietin significantly decreased both the iron-induced phosphorylated SMAD proteins and hepcidin mRNA. These results are in agreement with the recent observation that erythroferrone binds and inactivates the BMP6 protein. Administration of erythropoietin substantially increased the amount of erythroferrone and transferrin receptor 2 proteins in the spleen; pretreatment with iron did not influence the erythropoietin-induced content of these proteins. Erythropoietin-treated iron-deficient mice displayed smaller spleen size in comparison with erythropoietin-treated mice kept on a control diet. While the erythropoietin-induced increase in splenic erythroferrone protein content was not significantly affected by iron deficiency, the content of transferrin receptor 2 protein was lower in the spleens of erythropoietin-treated mice kept on iron-deficient diet, suggesting posttranscriptional regulation of transferrin receptor 2. Interestingly, iron deficiency and erythropoietin administration had additive effect on hepcidin gene downregulation in the liver. In mice subjected both to iron deficiency and erythropoietin administration, the decrease of hepcidin expression was much more pronounced than the decrease in phosphorylated SMAD protein content or the decrease in the expression of the SMAD target genes *Id1* and *Smad7*. These results suggest the existence of another, SMAD-independent pathway of hepcidin gene downregulation.

## Introduction

Iron is the oxygen-binding element in hemoglobin and is therefore indispensable for functional erythropoiesis. In contrast to other transition metals such as copper, molybdenum or cobalt, iron can not be actively excreted from the body, and its homeostasis is regulated only at the level of absorption of dietary iron in the duodenum [1]. The rate limiting process of iron absorption is the transfer of iron from the enterocyte into the bloodstream, which is mediated by the transmembrane iron exporter ferroportin. Ferroportin protein content at the enterocyte basolateral membrane is determined mainly by the circulating levels of the hepatocyte-derived peptide hepcidin, encoded by the *Hamp* gene [2,3,4].

The transcriptional control of hepcidin expression in the hepatocyte is maintained by several pathways, of which the bone morphogenetic proteins/SMAD pathway is the most studied [5]. The initial response of the liver to increased iron stores is the induction of bone morphogenetic protein 6 (BMP6), synthesized by non-parenchymal liver cells [6, 7, 8]. Next, the bone morphogenetic proteins (BMPs) bind, at the extracellular side of the hepatocyte plasma membrane, to heteromeric receptor complexes composed of type I receptors ALK2 and ALK3 and type II receptors BMPR2 and ACTR2A [9, 10]. This binding increases phosphorylation of the receptor-regulated SMAD1, SMAD5 and SMAD9 proteins inside the hepatocyte. The phosphorylated SMADs (pSMADs) bind with the common SMAD4, translocate to the nucleus, and ultimately increase the transcription of hepcidin. Clinical and experimental evidence indicates that the BMP/SMAD signaling is modulated by additional hepatocyte plasma membrane proteins such as hemojuvelin [11], HFE [12] and transferrin receptor 2 [13]. Whereas hemojuvelin functions as a co-receptor for the BMP proteins [14], the mode of action of HFE and transferrin receptor 2 (TFR2) is less clear. The physiological role of TFR2 is particularly puzzling, as several reports describe its expression and function in erythroid cells [15, 16, 17], in addition to its well-established presence on the hepatocyte plasma membrane.

Since erythropoiesis needs a constant supply of iron for the production of hemoglobin, the transcription of hepcidin is markedly decreased by an increase in erythropoietic activity. Very shortly after the discovery of hepcidin, it was demonstrated that liver hepcidin mRNA content dramatically decreases after administration of erythropoietin [18] and increases when erythropoiesis is suppressed by transfusion-induced polycythemia [19]. Importantly, the profound downregulation of *Hamp* expression by erythropoietin (EPO) is completely abolished when erythropoiesis is inhibited by irradiation [19] or administration of chemical inhibitors [20]. In contrast to the pathway mediating *Hamp* upregulation by iron, the pathway mediating *Hamp* downregulation by increased erythropoietic activity is much less understood. As early as 2006, it was proposed that *Hamp* expression is influenced by a substance released during erythropoiesis [20], and several candidate molecules, such as GDF15 [21] or TWSG1 [22] were described. In 2014, it was demonstrated that developing erythroblasts secrete a soluble protein, erythroferrone (ERFE, encoded by the *Fam132b* gene), which is transported in the bloodstream to the liver and mediates transcriptional downregulation of hepcidin expression [23]. According to the current concept, the primary function of erythroferrone is to act as a stress hormone that rapidly decreases hepcidin expression following acutely stimulated erythropoiesis [24]; however, it has also been proposed to contribute to low *Hamp* expression in conditions associated with chronic overproduction of EPO, such as β-thalassemias [25]. Interestingly, the effect of erythroferrone on *Hamp* gene downregulation is at least in part mediated through the BMP/SMAD pathway [26]; very recently, it has been proposed that the mode of action of erythroferrone involves its binding to the iron-inducible bone morphogenetic protein BMP6 [27].

As both iron overload and activated erythropoiesis apparently utilize the same signaling pathway to modulate hepcidin synthesis, it is probable that the net effect of these two opposing stimuli on *Hamp* expression is determined by their relative potency. Elucidation of the hierarchy between iron overload and erythropoietic activity on *Hamp* expression is of practical importance, as several diseases such as β-thalassemias, myelodysplastic syndromes, X-linked sideroblastic anemia or congenital dyserythropoietic anemia are characterized both by increased EPO levels and iron overload. Clinical evidence suggests that ineffective erythropoiesis associated with high circulating EPO levels efficiently downregulates *HAMP* expression even in the face of iron overload, since iron-overloaded patients with X-linked sideroblastic anemia continue to absorb iron [28]. On the other hand, the significant downregulation of murine *Hamp* expression by EPO can be reversed by iron pretreatment [29, 30], suggesting that erythroferrone is unable to efficiently suppress *Hamp* expression when the content of phosphorylated SMADs is high. Another support for the concept that high activity of the BMP/SMAD pathway overrides the erythropoiesis-related hepcidin downregulation comes from experiments with disruption of the *Tmprss6* gene, encoding the transmembrane serine protease matriptase-2. In humans, *TMPRSS6* mutations can result in iron-refractory iron deficiency anemia [31] characterized by inappropriately high hepcidin levels; in mice, lack of the functional protein leads to iron deficiency and marked microcytic anemia [32, 33]. Very probably, *TMPRSS6* cleaves a component of the hepatocyte SMAD signaling pathway and thus prevents hepcidin overexpression [34, 35]. In *Tmprss6*-mutated mice, pSMADs and the expression of their target genes *Id1* and *Smad7* are increased [36], and liver *Hamp* mRNA does not decrease even after repeated injections of EPO. Interestingly, the synthesis of erythroferrone protein is intact in the spleens of EPO-treated *Tmprss6*-mutated *mask* mice [37], which suggests that the defect in EPO signaling is located downstream of erythroferrone synthesis. Although it is well established that liver *Hamp* expression in *Tmprss6*-mutated mice does not respond to EPO treatment [30, 37, 38, 39], it is not yet known whether this indicates an absolute dominance of the BMP/SMAD signaling over erythroferrone signaling, or whether the absence of TMPRSS6 protein prevents the EPO-mediated *Hamp* gene downregulation by some other mechanism.

The purpose of the present study was to examine the effect of the crosstalk between BMP signaling and erythroferrone signaling by examining the effect of combined treatment with EPO and iron on liver *Hamp* expression, phosphorylated SMAD protein content and splenic ERFE and TFR2 protein content. We demonstrate that prolonged administration of EPO to C57BL/6J mice decreases the iron-induced *Hamp* expression, indicating that accelerated erythropoiesis can downregulate BMP/SMAD signaling even if the BMP/SMAD pathway is hyperactivated. We also show that the synthesis of ERFE protein in the spleen of EPO-treated mice is not affected by iron pretreatment, and that iron deficiency posttranscriptionally downregulates TFR2 protein content in the spleen of erythropoietin-treated mice. Finally, we demonstrate that iron deficiency and erythropoietic activity show an additive effect on the downregulation of *Hamp* gene expression. Overall, the results suggest that the attenuation of BMP/SMAD signaling by accelerated erythropoiesis or iron deficiency probably represents only one mechanism responsible for the downregulation of *Hamp* gene expression, and that other signaling pathway(s) might participate in this process. Further, the results of the combined treatment with iron and EPO can be viewed as an *in vivo* support for the recent concept that erythroferrone binds the iron-induced BMP6 protein.

## Materials and methods

### Animals and treatment

Animal experiments were approved by the Ministry of Education of Czech Republic, protocol MSMT-1461/2015-5, and by the Departmental Expert Comitee of The Czech Academy of Sciences for Approval of Animal Experiments, protocol no. 25/2015.

Male C57BL/6J mice (Anlab SRO, Prague, Czech Republic), age 2–3 months, were administered one intraperitoneal injection of iron dextran (Sigma Aldrich, 10 mg iron/mouse), control mice were administered 100 μl of phosphate-buffered saline (PBS). One week later, mice were treated by a daily intraperitoneal injection of 50 U of EPO (NeoRecormon 3000, Roche) in a total volume of 200 μl PBS for four consecutive days and euthanized by cervical dislocation 24 hours after the last injection; control mice received 200 μl of PBS.

For experiments with iron-deficient diet, 4-week old male C57BL/6J mice were placed on iron deficient diet (Altromin 1038, Lage, Germany, declared iron content 5 mg/kg) for 6 weeks, and then subjected to daily administration of EPO for four days as detailed above. Control animals were fed a standard laboratory diet (Altromin 1314, iron content approximately 200 mg/kg).

For experiments with *Tmprss6*-mutated *mask* mice, 2 to 3 month old male animals and their wild-type littermates were used. These mice are on a C57BL/6J background and were obtained from the Mutant Mouse Resource & Research Centers (USA).

Animal handling was optimized to minimize animal distress and suffering; mice were housed in groups of four in a standard-size cage, with free access to food and water. Animal status was checked daily. Blood for hematologic analyses was obtained under halothane anesthesia, animals were euthanized following anesthesia by cervical dislocation.

Hemoglobin and mean corpuscular volume (MCV) were determined on Mindray BC-5300Vet analyzer, hematocrit was determined by centrifugation in capillaries. Plasma iron content was determined by a commercial kit (Erba Lachema, Czech Republic), tissue iron was determined according to Torrance and Bothwell [40].

### Immunoblotting

For phosphorylated SMAD determinations, samples of liver (approximately 50 mg) were homogenized in 20 volumes of 50 mM Tris buffer, pH 8, containing 150 mM of sodium chloride, 1% of Igepal CA-630 detergent (Sigma Aldrich), 5 mM of EDTA, protease inhibitors (Complete Mini, Roche) and 1% of Phosphatase inhibitors cocktail 3 (Sigma Aldrich). After homogenization (3 x 10 sec by 6 mm Ultra Turrax homogenizer) samples were shaken for one hour at 4°C and then centrifuged at 12 000 g for 20 minutes; supernatant was aspirated and used for immunoblotting. Samples were mixed with loading buffer (Bio-Rad), heated for 10 min at 85° C and separated on 10% polyacrylamide gels. After blotting (X-cell Sure Lock, Invitrogen, USA), the membranes were blocked for one hour in filtered 5% skimmed milk (Regilait, France) and incubated overnight with Abcam ab92698 anti pSMAD 5 antibody (1:1000). This antibody has been previously reported to cross-react with pSMAD 1 [26].

For ERFE and TFR2 determinations, spleen microsomes (membranes obtained by ultracentrifugation of the post-mitochondrial supernatant) were used. The study focused on splenic ERFE synthesis, rather than on bone marrow ERFE synthesis, because the spleen is regarded as an important organ in murine stress erythropoiesis [41]; in addition, the use of spleen provided enough material for the isolation of microsomes, which facilitate ERFE and TFR2 protein detection [37]. Samples of spleen (approximately 50 mg) were homogenized in 20 volumes of 10 mM HEPES, pH 7.4, 5 mM EDTA, protease inhibitors and 250 mM sucrose. The homogenate was centrifuged at 8 000 g for 15 min and the aspirated supernatant was then centrifuged at 100 000 g for one hour to obtain the microsomal pellet. The pellet was washed by another centrifugation at 100 000 g and resuspended in 100 μl of 2% SDS, buffered with 25 mM of ammonium bicarbonate. After electrophoresis and blotting, the immunoblot membrane was incubated with Santa Cruz Sc-246567 polyclonal anti-myonectin antibody (discontinued in 2016), dilution 1:100, for the detection of ERFE; or with Alpha Diagnostics International TFR21-A antibody, batch 601617A-1.5-P, dilution 1:500, for the detection of TFR2. Both antibodies are specific for their targets (reference [37], S1 Fig); the anti-TFR2 antibody detects the intracellular terminus of the protein. Anti-GAPDH (Sigma G9545; 1:30 000) antibody was used for loading control detection. Secondary antibodies (anti-rabbit, 711-036-152, 1:40 000 and anti-goat, 705-036-147, 1:40 000) were obtained from Jackson Immunoresearch. Secondary antibodies were diluted in 5% milk and membranes were incubated for two hours. Proteins were visualized after reaction with LumiGLO (Cell Signaling Technology) on ChemiDoc MP imaging system from Bio-Rad, band densities were calculated using LI-COR software.

### Real-time PCR

RNA was isolated from tissue samples stored in RNA-Later (Sigma Aldrich) using Qiagen RNeasy Plus kit; for the determination of bone marrow gene expression, marrow from one femur was directly aspirated in the RNeasy homogenization buffer. RNA was reverse transcribed using RevertAid kit (Thermo Scientific). Real-time PCR was performed on a Bio-Rad IQ5 instrument, primer sequences are given in S1 Table. Primers were designed using Primer 3 software, *Smad7* primer sequence was described previously by Kautz *et al*. [42]. Results are expressed as Δ CT values relative to *Actb* expression (Δ CT = *Actb* cycle threshold–target gene cycle threshold); the higher the graphed Δ CT value, the higher the expression.

### Statistical analysis

Values are expressed as mean ± SD. Data were analyzed using one-way ANOVA, followed by Tukey multiple comparison test.

## Results

### Repeated administration of EPO decreases the iron-induced phosphorylated SMAD content in the liver of C57BL/6J mice

**Fig 1:**
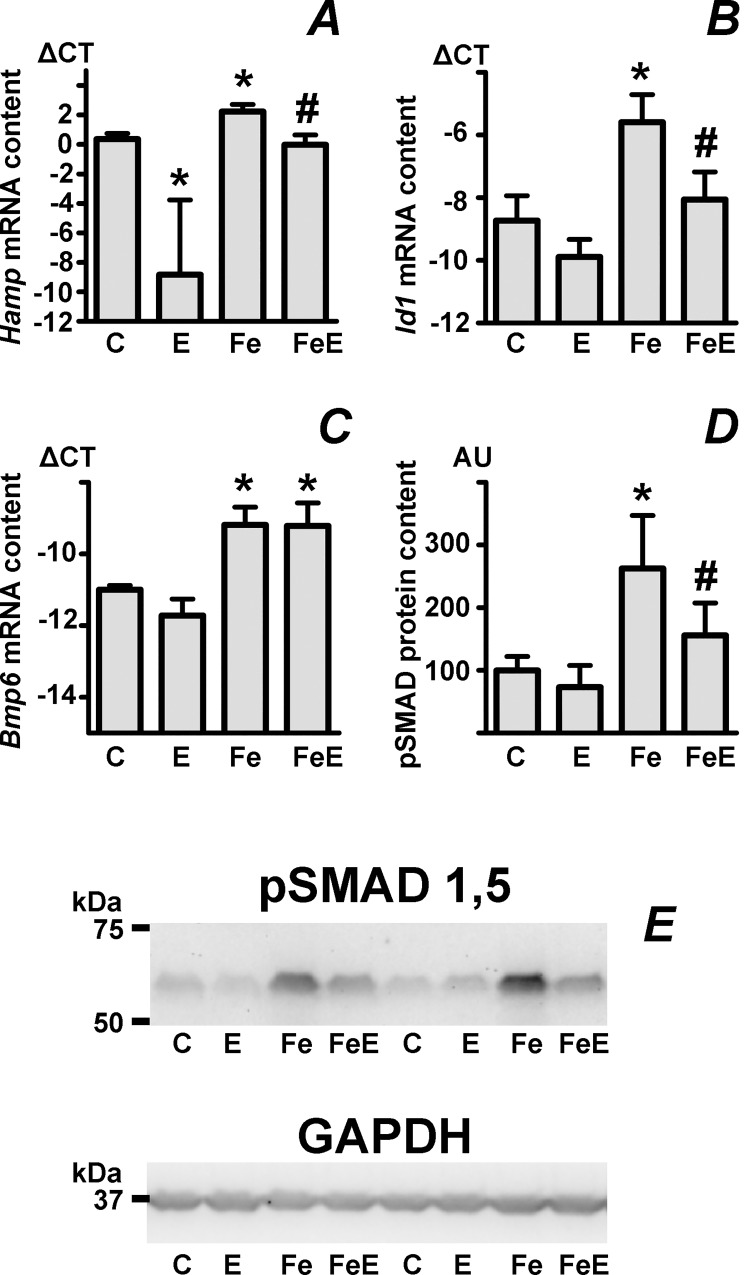
The effect of combined treatment with iron and EPO on *Hamp* expression and pSMAD signaling. A-C: Liver *Hamp*, *Id1* and *Bmp6* mRNA content, expressed as ΔCT relative to *Actb* mRNA content. Male C57BL/6 mice were pretreated with one intraperitoneal injection of iron dextran (500 mg/kg) one week before EPO administration (50 U/mouse daily on four consecutive days). ΔCT is calculated as *Actb* CT minus target CT. n = 5 for *Hamp* and *Id1*, n = 3 for *Bmp6*. D: Calculated phosphorylated SMAD 1,5 (pSMAD 1,5) protein content relative to the control group, expressed in arbitrary units (AU). n = 8. E: Immunoblot of pSMAD 1,5 protein in mouse liver homogenates. GAPDH is used as loading control. Column abbreviations: C: Control group, E: EPO-treated group, Fe: Iron dextran-pretreated group, FeE: Iron dextran-pretreated group administered EPO. Asterisks denote statistically significant difference from control group, hash tag denotes statistically significant difference between Fe and FeE groups.

Original experiments addressing the interaction between iron and EPO on hepcidin expression in mice demonstrated that repeated administration of EPO for four days decreased the elevated expression of *Hamp* induced by administration of iron-rich diet, as well as the iron-induced liver pSMAD protein content [43]. However, results from experiments with short term (15 h) EPO administration indicated no effect of EPO on iron-induced *Hamp* mRNA content [30]. To re-evaluate the effect of combined treatments on liver *Hamp* expression and pSMAD protein content, iron-overloaded mice were treated with EPO for four days, resulting in the expected changes in hematologic parameters (S2 Fig) and tissue iron content (S2 Table). As expected [42], iron pretreatment increased the expression of *Hamp*, *Id1* and *Bmp6* (Fig 1A, 1B and 1C). Data from Fig 1A and 1B confirm that, even in the presence of significant iron overload caused by iron injection, increased *Hamp* and *Id1* expression can be partially downregulated by repeated administration of EPO (columns Fe versus FeE). Also, the PCR data confirms the previous observations [29, 30] that iron pretreatment very efficiently attenuates the dramatic EPO-induced downregulation of *Hamp* expression (Fig 1A, column E versus FeE). In agreement with the observed effect of EPO on iron-induced *Hamp* mRNA content (Fig 1A, column Fe versus FeE), the immunoblot presented in Fig 1E clearly demonstrates that EPO significantly downregulates the high amount of pSMADs induced by a single injection of iron. Additional blots demonstrating the effect of EPO on iron-induced pSMAD protein content are shown in S3 Fig; values plotted in graphs are shown in S3 Table.

### Phosphorylated SMAD protein content is higher in iron-treated C57BL/6J mice than in *mask* mice; nevertheless, it can be downregulated by EPO

**Fig 2:**
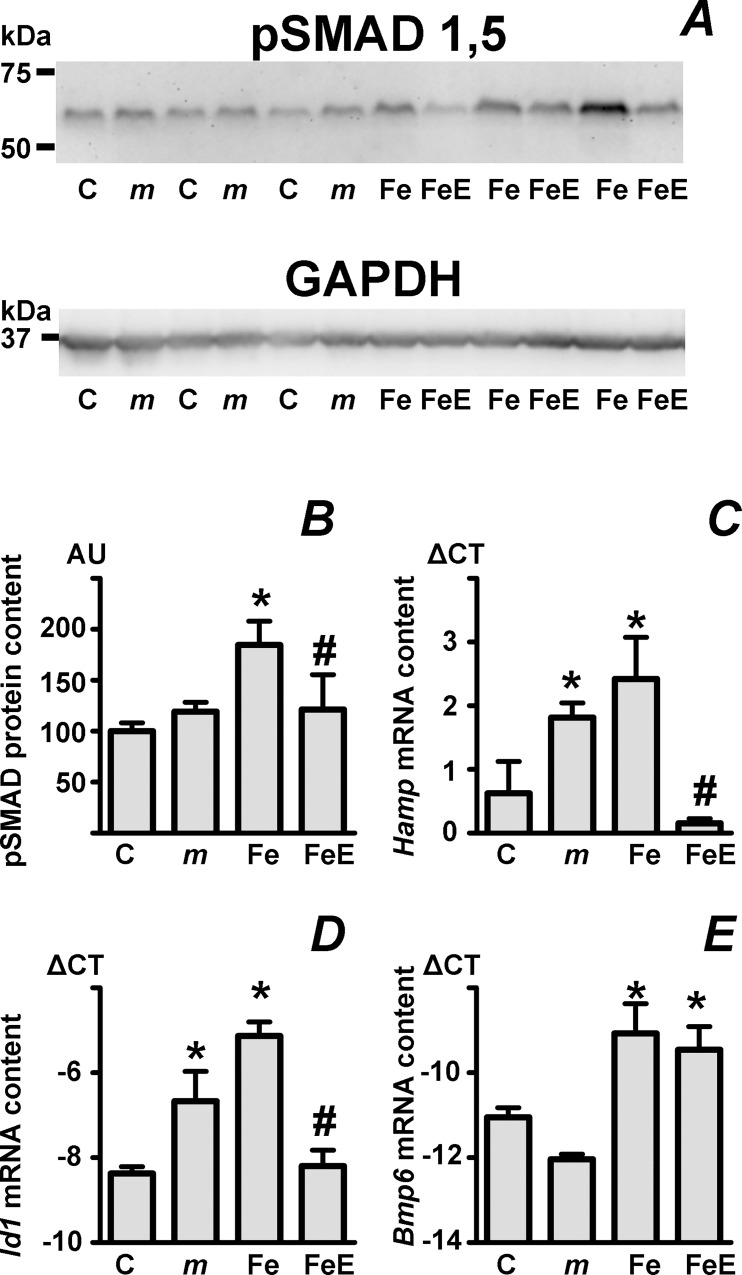
Comparison of BMP/SMAD signaling between *mask* mice and iron-treated C57BL/6 mice. A: Immunoblot of phosphorylated SMAD 1 and 5 protein (pSMAD 1,5) in liver homogenates prepared from C57BL/6 mice (C), *mask* mice (*m*), iron-pretreated C57BL/6 mice (Fe) and C57BL/6 mice treated with the combination of iron and EPO (FeE). Treatment details as in Fig 1, GAPDH is used as loading control. B: Calculated pSMAD 1,5 protein content from panel A expressed in arbitrary units (AU) relative to the control group. n = 3. C-E: Liver *Hamp*, *Id1* and *Bmp6* mRNA content, expressed as ΔCT relative to *Actb* mRNA content, from liver samples analyzed in panel A. Asterisk denotes statistically significant difference from control group, hash tag denotes statistically significant difference between Fe and FeE groups, n = 3.

The complex nature of the interactions between BMP/SMAD signaling and ERFE signaling is illustrated by the puzzling *Hamp* gene regulation in *mask* mice. These mice are characterized by over-activation of the pSMAD pathway [36], resulting in *Hamp* gene upregulation and iron deficiency. It is very well established that the upregulated *Hamp* mRNA content in *mask* mice can not be lowered by repeated administration of EPO [30, 37, 38, 39], leading to the conclusion that over-activation of the BMP/SMAD pathway prevents the action of ERFE [30, 44]. Results presented in Fig 2 indicate that although the BMP/SMAD pathway is over-activated in *mask* mice (Fig 2D), the liver content of phosphorylated SMAD proteins in these mice (Fig 2A and 2B), as well as liver *Hamp* and *Id1* mRNA content (Fig 2C and 2D), does not exceed values seen in iron-treated C57BL/6J mice. Intriguingly, the high phosphorylated SMAD protein content in iron-treated C57BL/6J mice can be efficiently downregulated by administration of EPO (Fig 2A and 2B), whereas in *mask* mice repeated EPO administration has no effect on phosphorylated SMAD protein content (S4 Fig). The results support the recent concept that high ERFE levels attenuate the BMP6-induced pSMAD signaling [27], rather than basal pSMAD signaling.

### Iron status does not influence the EPO-induced synthesis of erythroferrone protein in the spleen

**Fig 3:**
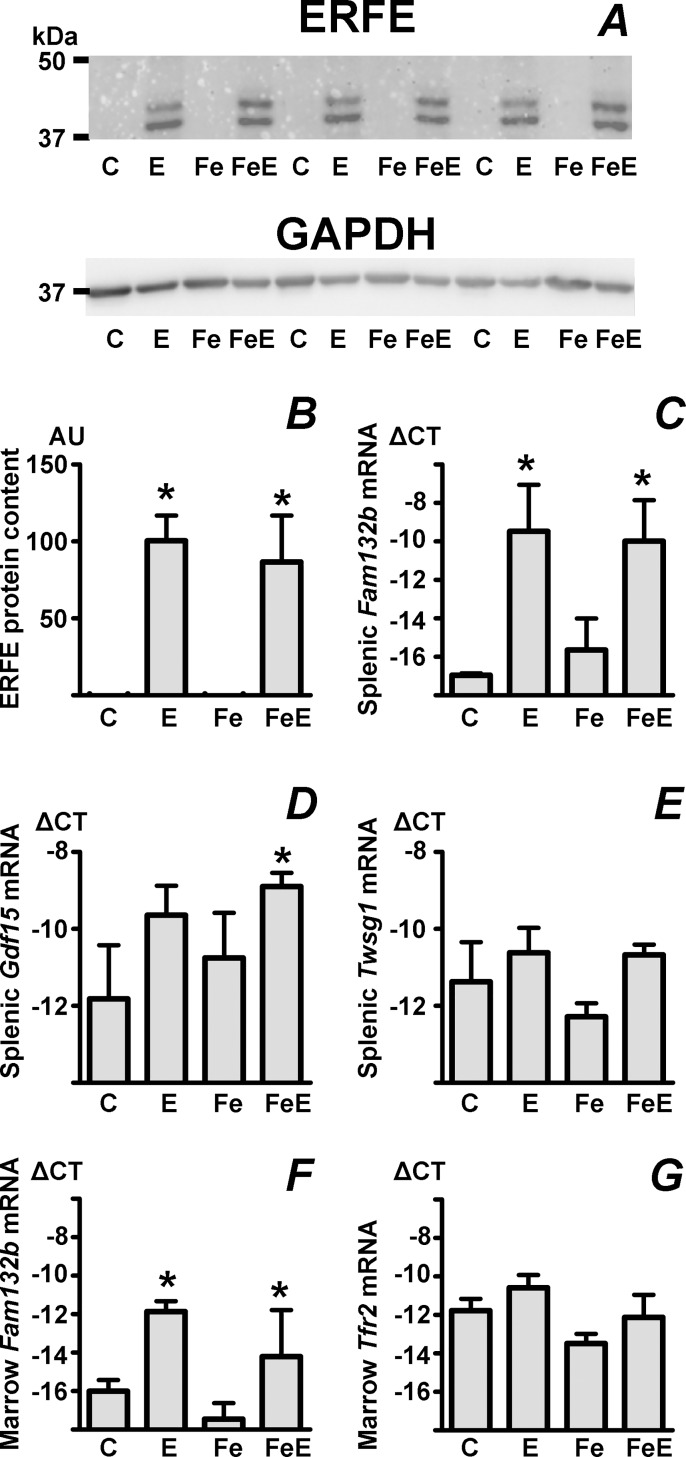
Iron overload does not influence the EPO-induced expression of erythroferrone protein in mouse spleen. A: Immunoblot of erythroferrone (ERFE) in spleen microsomes prepared from control C57BL/6 mice (C), EPO-treated mice (E), iron-pretreated C57BL/6 mice (Fe) and C57BL/6 mice treated with the combination of iron and EPO (FeE). Treatment details as in Fig 1, GAPDH is used as loading control. B: Calculated ERFE protein content, expressed in arbitrary units (AU) relative to the EPO-treated group, n = 6. C-E: Splenic *Fam132b*, *Gdf15* and *Twsg1* mRNA content, n = 4. F and G: *Fam132b* and *Tfr2* mRNA content in bone marrow, n = 3. Target mRNA content is expressed relative to *Actb* mRNA content, ΔCT is calculated as *Actb* CT minus target CT. Asterisks denote statistically significant differences from control groups.

A theoretical explanation for the marked effect of iron pretreatment on EPO-downregulated *Hamp* mRNA levels (columns E and FeE in Fig 1A) could be that iron overload negatively influences the expression of ERFE protein. However, results presented in Fig 3A and 3B show that ERFE protein synthesis in the spleen of EPO-treated mice is not affected by iron pretreatment. This data confirms similar observations in rats [45], indicating that the regulation of ERFE expression is primarily transcriptional and independent of iron status. In contrast to the dramatic induction of *Fam132b* mRNA (Fig 3C), the effect of EPO on splenic *Gdf15* and *Twsg1* mRNA was only modest (Fig 3D and 3E), confirming the concept that these proteins probably do not participate in the regulation of *Hamp* gene expression under physiological conditions [44]. Similarly to the induction of *Fam132b* in the spleen (Fig 3C), repeated administration of EPO also increased *Fam132b* mRNA content in the bone marrow (Fig 3F).

### Iron deficiency decreases the EPO-induced synthesis of TFR2 protein in the spleen

**Fig 4:**
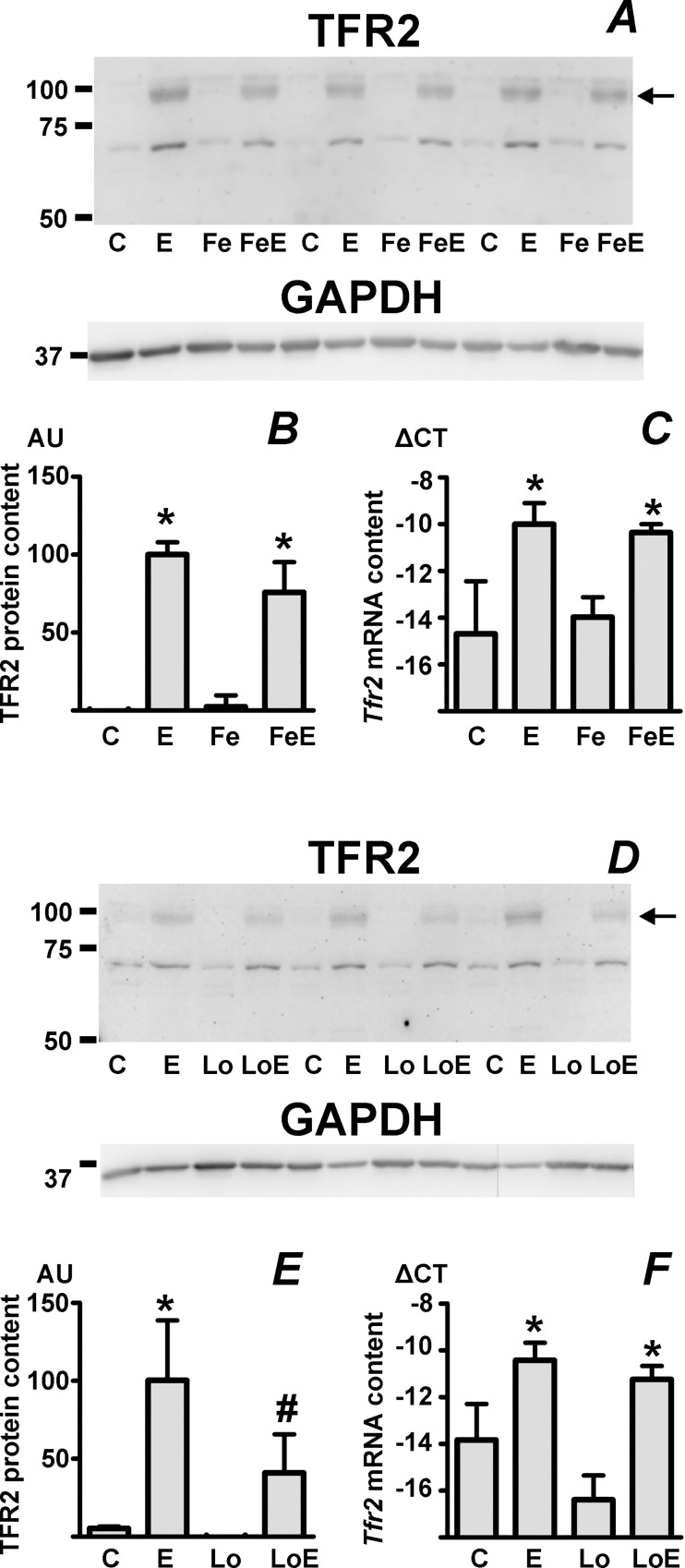
Iron deficiency decreases the EPO-induced expression of TFR2 protein in mouse spleen. A: Immunoblot of transferrin receptor 2 (TFR2) in spleen microsomes prepared from control C57BL/6 mice (C), EPO-treated mice (E), iron-pretreated C57BL/6 mice (Fe) and C57BL/6 mice treated with the combination of iron and EPO (FeE). GAPDH is used as loading control, arrow indicates the TFR2 band. B: Calculated TFR2 protein content, expressed in arbitrary units (AU) relative to the EPO-treated group. Treatment details as in panel A. Asterisk denotes statistically significant difference from control group, n = 6. C: Splenic *Tfr2* mRNA content, expressed as ΔCT relative to *Actb* mRNA content. Asterisk denotes statistically significant difference from control group, n = 3. D: Immunoblot of transferrin receptor 2 (TFR2) in spleen microsomes prepared from control C57BL/6 mice (C), EPO-treated mice (E), C57BL/6 mice kept on an iron-low diet for six weeks (Lo) and C57BL/6 mice kept on an iron-low diet and treated with erythropoietin (LoE). GAPDH is used as loading control. E: Calculated TFR2 protein content, expressed in arbitrary units (AU) relative to the EPO-treated group. Treatment details as in panel D. Asterisk denotes statistically significant difference from control group, hash tag denotes statistically significant difference between E and LoE groups, n = 6. F: Splenic *Tfr2* mRNA content, expressed as ΔCT relative to *Actb* mRNA content. Treatment details as in panel D. Asterisk denotes statistically significant difference from control group, n = 3.

TFR2 is a hepatocyte membrane protein [13] whose mutations can lead to hemochromatosis [17]. In addition to hepatocytes, TFR2 is also expressed in erythroid cells, where it possibly influences EPO sensitivity [15] and modulates the inefficient erythropoiesis observed in β-thalassemia [46]. Interestingly, at both mRNA and protein level, splenic TFR2 is dramatically induced by EPO treatment [37]. Since splenic TFR2 protein could, by shedding of a soluble TFR2 form [47], theoretically influence the synthesis of hepcidin through interaction with the BMP pathway [48], and since the expression of liver TFR2 protein is posttranscriptionally regulated by iron status [49, 50], it was of interest to examine whether splenic TFR2 induction by EPO could be also modulated by iron overload or iron deficiency. Data presented in Fig 4A and 4B show that the EPO-induced splenic TFR2 protein content is not significantly affected by iron pretreatment. This is in contrast to TFR2 regulation in the liver, where iron overload significantly increases the amount of the protein (S5 Fig). In agreement with our previously published data [37], EPO treatment increased *Tfr2* mRNA content in the spleen (Fig 4C); the EPO-induced increase in bone marrow *Tfr2* mRNA content was less pronounced and did not reach statistical significance (Fig 3G). Although splenic TFR2 protein content was not influenced by iron overload (Fig 4A and 4B), iron deficiency partially attenuated the EPO-induced increase in splenic TFR2 protein (Fig 4D and 4E). EPO-induced splenic *Tfr2* mRNA content was not influenced by iron deficiency (Fig 4F).

### Administration of EPO to iron-deficient mice further decreases *Hamp* gene expression

**Fig 5:**
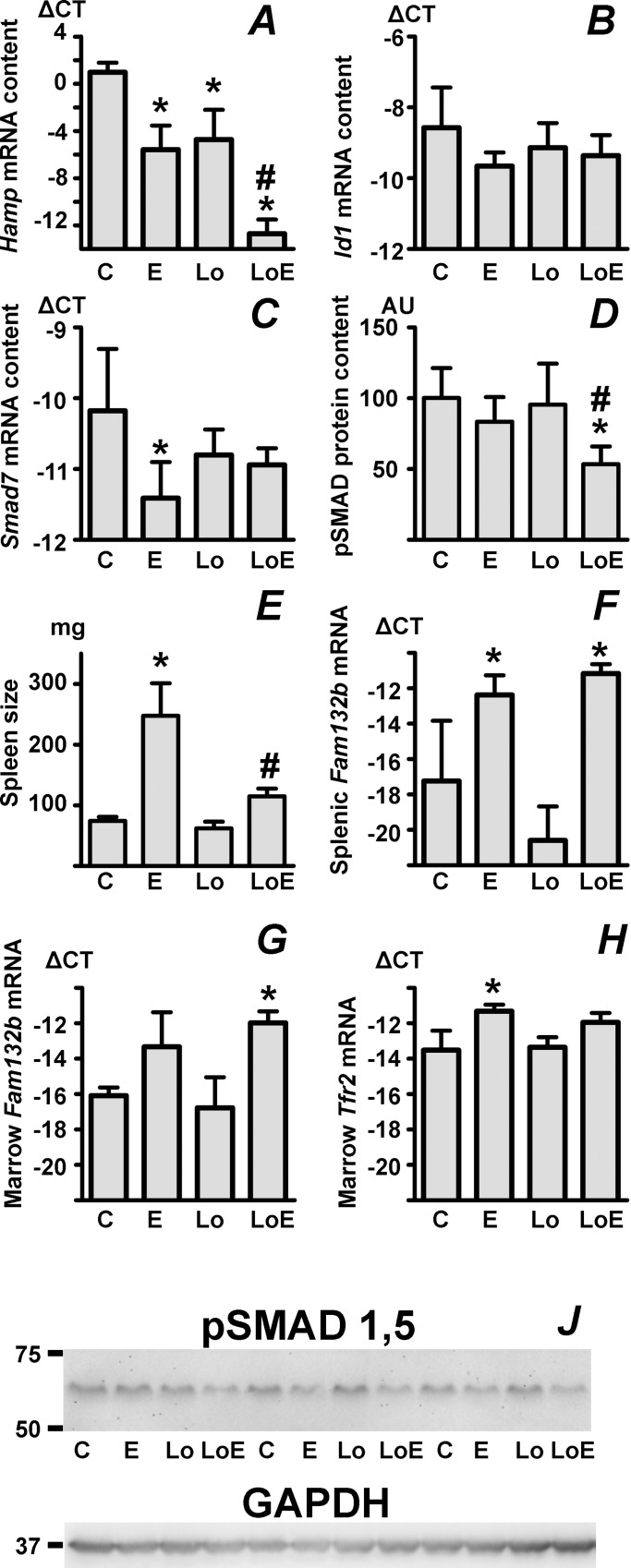
Iron deficiency and EPO administration display additive effect on liver *Hamp* gene expression. A-C: Liver *Hamp*, *Id1* and *Smad7* mRNA content in samples prepared from control C57BL/6 mice (C), EPO-treated mice (E), mice kept on a low-iron diet for six weeks (Lo) and mice kept on a low-iron diet for six weeks and then treated with four daily doses of erythropoietin (LoE). ΔCT is calculated as *Actb* CT minus target CT, n = 4. D: Calculated phosphorylated SMAD 1 and 5 (pSMAD 1,5) protein content, expressed in arbitrary units (AU) relative to the control group, n = 4. E: Spleen size in the experimental groups, n = 4. F: Splenic *Fam132b* mRNA content, n = 4. G and H: Bone marrow *Fam132b* and *Tfr2* mRNA content, n = 3. J: Immunoblot of pSMAD 1,5 protein in liver homogenates, experimental groups as in panel A. GAPDH is used as loading control. Asterisks denote statistically significant difference from control group, hash tags denote statistically significant difference between Lo and LoE groups.

In order to study the regulation of *Hamp* expression in conditions of iron deficiency, four-week old male C57BL/6J mice were placed on control or iron-deficient diet for 6 weeks. This treatment decreased the mean cell volume, but did not yet cause iron deficiency anemia (S1 Fig). As shown in Fig 5A, treatment with iron-deficient diet significantly decreased liver *Hamp* expression, although it did not increase splenic *Fam132b* expression (Fig 5F, column C versus Lo). Liver iron content was significantly decreased, while plasma iron content was not significantly changed (S2 Table). Administration of EPO to iron-deficient mice resulted in further profound decrease in *Hamp* expression (Fig 5A, column LoE). Plasma iron content in EPO-treated mice kept on control or iron-deficient diets was significantly decreased (S2 Table). As expected, administration of EPO increased the spleen size (Fig 5E). The EPO-induced increase in spleen size was clearly dependent on iron availability, as the spleens from EPO-treated mice kept on low-iron diet were significantly smaller than spleens from EPO-treated mice kept on control diet (Fig 5E); nevertheless, the EPO-induced increase in splenic *Fam132b* mRNA and *Tfr2* mRNA content was not influenced by iron deficiency (Fig 5F and Fig 4F). Similarly to the spleen, administration of EPO also induced *Fam132b* and *Tfr2* mRNA in the bone marrow (Fig 5G and 5H), the effect of EPO on marrow *Tfr2* expression was less pronounced than in the spleen. Interestingly, although the effect of the combined treatment on *Hamp* expression was dramatic (Fig 5A, column LoE), the changes in *Id1* mRNA content (Fig 5B), *Smad7* mRNA content (Fig 5C) and liver pSMAD content (Fig 5D and 5J) were only moderate, suggesting that in this experimental setting, the downregulation of *Hamp* expression is only partially dependent on the attenuation of BMP/SMAD signaling. Additional immunoblot showing the effect of the combined treatment (low-iron diet and EPO) on pSMADs is included as S6 Fig.

## Discussion

Hepcidin, the master regulator of systemic iron metabolism, is known to respond to two major stimuli: Iron overload increases *Hamp* gene expression, whereas accelerated erythropoiesis decreases *Hamp* expression. According to current concepts, the effect of iron is mediated by increased synthesis of the BMP6 protein, whereas the effect of EPO is partly mediated by increased synthesis of erythroferrone [23, 24, 44], and partly by the EPO-induced redistribution of iron [19, 20]. The molecular basis of the crosstalk between the iron-induced and EPO-induced pathways was first investigated in 2009, when Huang *et al*. demonstrated that repeated doses of EPO, administered over four days, can inhibit the increase of *Hamp* mRNA induced by dietary iron, and that this effect correlates with the liver content of phosphorylated SMADs [43]. During further studies on the interplay of the two stimuli, we have reported that, *vice versa*, a high dose of iron can to a very significant extent prevent the downregulation of *Hamp* expression elicited by repeated doses of EPO [29]. Thus, in the four-day experimental setting, both stimuli are able to partially antagonize the effect of the other. After the discovery of erythroferrone it was shown that iron-pretreatment blunts the EPO-induced downregulation of *Hamp* expression in a short-term (15 h) experiment [30], but, in contrast to prolonged EPO administration, EPO in this short-term setting did not attenuate the elevated *Hamp* expression induced by iron. Based on these observations, it has been postulated that erythroferrone can not efficiently downregulate *Hamp* expression when the BMP/SMAD pathway is hyperactivated [30, 44]. However, results presented in this study indicate that, in agreement with the original data by Huang *et al* [43], EPO administration can to some extent decrease the iron-induced increase in phosphorylated SMAD proteins, as well as the iron-induced *Hamp* and *Id1* expression. These data can be interpreted as an *in vivo* support for the very recent proposal that erythroferrone attenuates signaling through the BMP/SMAD pathway by binding of the BMP6 protein [27]. As can be seen in Fig 1C, liver *Bmp6* expression is induced to the same extent both in the iron-treated group and the iron plus EPO-treated group, but *Hamp* mRNA content, *Id1* mRNA content and pSMAD protein content in the iron plus EPO-treated group are significantly reduced (Fig 1A, 1B and 1D, columns Fe versus FeE). The recent hypothesis proposed by Arezes *et al*. [27], namely that the activity of the BMP/SMAD signaling pathway is blunted by inactivation of the secreted BMP6 protein by circulating erythroferrone, is in agreement with these results.

One of the puzzling features related to the crosstalk between erythropoiesis and iron status is the total lack of effect of EPO on erythropoiesis in mice with mutations in the *Tmprss6* gene. Lack of matriptase-2, the product of the *Tmprss6* gene, hyperactivates the BMP/SMAD signaling pathway by a yet not completely understood mechanism [35], and it has been suggested that the hyperactivated BMP/SMAD signaling is refractory to inhibition by erythroferrone [44]. However, as can be seen in Fig 2, EPO administration can partially downregulate BMP/SMAD signaling in iron-treated C57BL/6 mice, despite the fact that the iron-induced phosphorylated SMAD protein content is higher in iron-treated C57BL/6 mice than in *Tmprss6*-mutated *mask* mice. These results can be again interpreted as an indirect support for the recently suggested interaction between BMP6 and erythroferrone [27]. If erythroferrone indeed binds and inactivates the BMP6 protein, then it can not be expected to effectively inhibit *Hamp* expression in *Tmprss6*-mutated mice, as these mice express even less *Bmp6* mRNA than untreated wild-type mice (Fig 2E).

As early as 2006 it was proposed that erythroblasts could produce a secreted protein which affects *Hamp* expression in the hepatocyte [20]. Possible candidates include GDF15 [21], TWSG1 [22], and, most recently, ERFE [23]. It has been already convincingly demonstrated that murine *Fam132b* mRNA content (encoding ERFE) significantly increases in the bone marrow and spleen of EPO-treated mice [23]; however, there is only limited information on the possible modulation by iron overload or iron deficiency of the EPO-induced ERFE synthesis at the protein level. Results presented in this study demonstrate that iron overload or iron deficiency did not affect ERFE protein induction in the spleen. Therefore, the very significant effect of iron on EPO-downregulated *Hamp* mRNA content (Fig 1A, columns E versus FeE) can not be explained by altered ERFE protein synthesis. Much more likely, the potent effect of iron is related to the recent observation by Mirciov *et al*. [51], who described rapid influence of changes in diferric transferrin concentration on liver pSMAD content and EPO-induced *Hamp* expression.

Based on results reported by several recent papers [16, 17, 46], the response of *Hamp* to EPO could be also modulated by erythroid-specific synthesis of TFR2. In contrast to ERFE, which is synthesized almost exclusively in erythroblasts, TFR2 is mainly produced by hepatocytes; however, its mRNA can be detected in erythroid cells [13, 52], and its synthesis is significantly increased in EPO-treated spleen at both mRNA and protein level [37]. Increased erythroid synthesis of TFR2 could theoretically either positively [15] or negatively [53] affect the responsiveness of erythroid cells to EPO. Alternatively, increased splenic TFR2 synthesis could directly influence hepatocyte *Hamp* expression through the release of a soluble TFR2 form [47]. Although, based on *in vitro* data, this mode of action is regarded as unlikely [47], the recent demonstration that the extracellular domain of TFR2 can bind BMP proteins [48] raises new and interesting perspectives about the possible role of shed TFR2 in the modulation of BMP signaling. Results presented in this study confirm that EPO treatment markedly induces splenic TFR2 protein synthesis, and demonstrate that the EPO-induced splenic TFR2 protein content is apparently not influenced by iron overload, which is in contrast to the marked effect of iron on TFR2 protein content in the liver [49, 50]. On the other hand, iron deficiency partially attenuated the induction of splenic TFR2 protein by EPO, suggesting that both liver and splenic TFR2 proteins are to some extent posttranscriptionally regulated by iron availability. Overall, this part of the study did not identify any obvious effect of iron pretreatment on the transcriptional regulation of the four examined genes, *Fam132b*, *Gdf15*, *Twsg1* and *Tfr2*, suggesting that iron pretreatment attenuates the EPO-induced *Hamp* downregulation mainly by its effect on transferrin saturation and SMAD phosphorylation [51], rather than by modulation of the synthesis of candidate erythroid regulators. Regarding the possible role of the shed extracellular domain of erythroid TFR2 on BMP-dependent signaling [48], further studies, depending on reliable antibodies against the extracellular part of TFR2, will be needed to clarify this issue.

Although the effect of EPO on *Hamp* expression has during the past years been attributed mainly to erythroferrone, which is currently regarded as the most important physiological regulator linking erythropoietic activity with iron absorption [44], recent data unexpectedly indicate that erythroferrone is dispensable for *Hamp* gene downregulation if EPO is administered for longer time intervals [24]. This observation is in agreement with the original concept that ERFE is primarily a stress hormone that rapidly decreases hepcidin expression following acutely stimulated erythropoiesis [23]. The fact that *Fam132b*-deficient mice decrease hepcidin expression as efficiently as wild-type mice following chronic administration of EPO [24] suggests that one of the main factors participating in EPO-induced *Hamp* gene downregulation could be the decrease in transferrin saturation caused by increased flux of available iron into the erythroid compartments, as originally proposed by Vokurka *et al*. [19]. There is relatively little information on *Hamp* gene expression in mice subjected both to EPO treatment and iron deficiency; in particular, it is not established to what extent the administration of EPO will influence the already low *Hamp* mRNA content in iron-deficient mice. To examine this combined regulation, we administered four doses of EPO to mice kept for six weeks since weaning on an iron deficient diet; this pretreatment decreased the mean cell volume, but did not yet cause iron deficiency anemia. The results clearly indicate that in this experimental setting, the effects of iron deficiency and EPO administration on *Hamp* expression are additive–apparently, feeding of the iron deficient diet decreases liver iron content, whereas administration of EPO decreased plasma iron content. Since the modest decrease in phosphorylated SMADs observed in EPO-treated mice kept on iron deficient diet did not parallel the profound decrease in *Hamp* expression, the results point to the possibility that decreased signaling through the BMP/SMAD pathway represents only one of the mechanisms which downregulate *Hamp* expression. This conclusion is in agreement with the recent observation that mice with hepatocyte-specific disruption of SMAD1 and SMAD5 synthesis are still able to downregulate *Hamp* expression when placed for three weeks on an iron-deficient diet [26]. Although it has been reported that *Hamp* expression is regulated by several signaling pathways in addition to the BMP/SMAD pathway [54, 55], the BMP/SMAD pathway has so far received the most attention, since its participation in *Hamp* gene upregulation by iron is relatively well understood and well established [5, 14]. However, the recently published results from studies with SMAD-deficient mice [26] suggest that *Hamp* gene downregulation by iron deficiency could be also dependent on other pathways, which have so far not been completely characterized. The discrepancy between the dramatic decrease in *Hamp* expression (Fig 5A) and the expression of *Id1* (Fig 5B), which is regarded as a marker of BMP/SMAD signaling [56, 57] apparently supports this concept.

## Conclusions

In conclusion, the study demonstrates that prolonged administration of EPO to C57BL/6 mice decreases the iron-induced hepcidin expression, indicating that accelerated erythropoiesis can downregulate BMP/SMAD signaling even if the BMP/SMAD pathway is hyperactivated, and indirectly supporting the concept that erythroferrone can bind and inactivate the BMP6 protein. It provides new data on the induction of splenic ERFE and TFR2 by EPO, suggesting that the induced levels of these proteins are not further posttranscriptionally regulated by iron overload, but that the EPO-induced splenic TFR2 protein levels are decreased by iron deficiency. It further shows that iron deficiency and EPO administration have an additive effect on the downregulation of liver *Hamp* expression. Finally, in experiments examining the combined effect of EPO-treatment and iron deficiency, the discrepancy between the limited decrease of liver pSMAD content and *Id1* mRNA content on the one hand and the dramatic decrease in *Hamp* mRNA content on the other strongly suggest the existence of another, SMAD-independent pathway of *Hamp* gene downregulation.

## Supporting information

S1 Fig TFR2 antibody validation. (DOC)

S2 Fig Hematologic parameters of animals used in the experiments. (DOC)

S3 Fig Additional immunoblots to Fig 1. (DOC)

S4 Fig Erythropoietin does not decrease phosphorylated SMAD 1,5 protein content in *mask* mice. (DOC)

S5 Fig Iron treatment increases TFR2 protein content in liver microsomes. (DOC)

S6 Fig Additional immunoblot to Fig 5. (DOC)

S1 Table List of primers used for PCR analysis. (DOC)

S2 Table Iron status of animals used in the experiments. (DOC)

S3 Table Values used to build graphs and tables. (XLSX)
