# Supplementary material for: Effect of stimulated erythropoiesis on liver SMAD signaling pathway in iron-overloaded and iron-deficient mice
Source: PLoS One. 2019 Apr 8;14(4):e0215028. doi: 10.1371/journal.pone.0215028 (PMC6453526; doi:10.1371/journal.pone.0215028)
Supplement: S1 Fig — (DOC) [file pone.0215028.s001.doc]

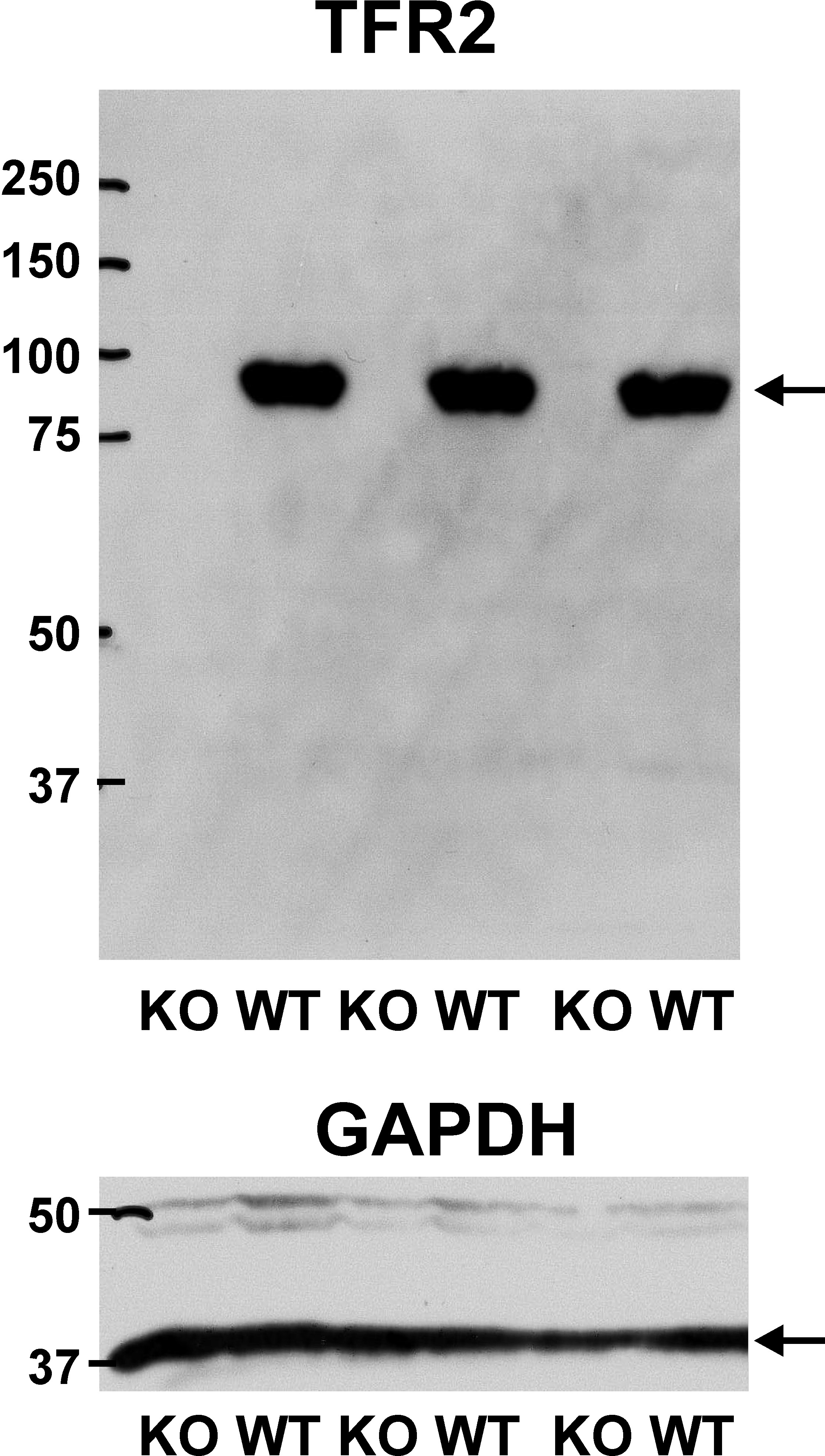


**S1 Fig. TFR2 antibody validation.** Immunoblot of liver microsomes prepared from *Tfr2*-/- mice (KO) and *Tfr2*+/+ mice (WT). Alpha Diagnostics International TFR21-A antibody, batch 601617A-1.5-P, dilution 1:500, was used for TFR2 detection. 8% polyacrylamide gel, loading 40 μg/well. GAPDH is used as loading control, arrows indicate the specific bands.
