# Supplementary material for: Effect of stimulated erythropoiesis on liver SMAD signaling pathway in iron-overloaded and iron-deficient mice
Source: PLoS One. 2019 Apr 8;14(4):e0215028. doi: 10.1371/journal.pone.0215028 (PMC6453526; doi:10.1371/journal.pone.0215028)
Supplement: S2 Fig — (DOC) [file pone.0215028.s002.doc]

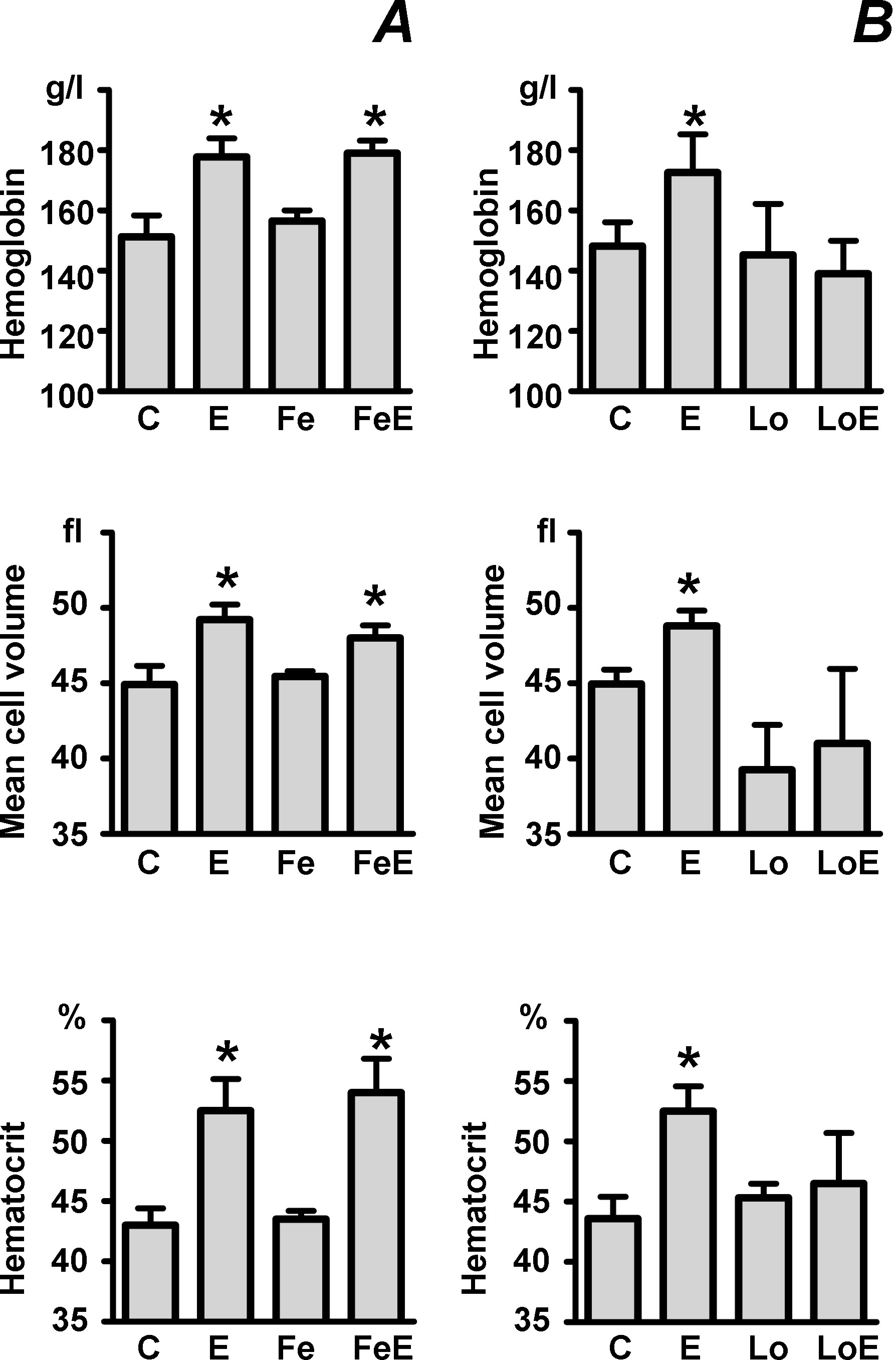


**S2 Fig. Hematologic parameters of animals used in the experiments.** A: Hematologic parameters in mice treated with a combination of iron and erythropoietin. B: hematologic parameters in mice treated with a combination of low-iron diet and erythropoietin. Tratment details as in Materials and Methods. C: Control mice; E: EPO-treated mice; Fe: Iron-treated mice; FeE: Mice treated with a combination of iron and EPO; Lo: Mice kept on a low-iron diet; LoE: Mice kept on a low-iron diet and treated with EPO. Asterisks denote statistically significant difference from control group, n>3.
