# Supplementary material for: Effect of stimulated erythropoiesis on liver SMAD signaling pathway in iron-overloaded and iron-deficient mice
Source: PLoS One. 2019 Apr 8;14(4):e0215028. doi: 10.1371/journal.pone.0215028 (PMC6453526; doi:10.1371/journal.pone.0215028)
Supplement: S3 Fig — (DOC) [file pone.0215028.s003.doc]

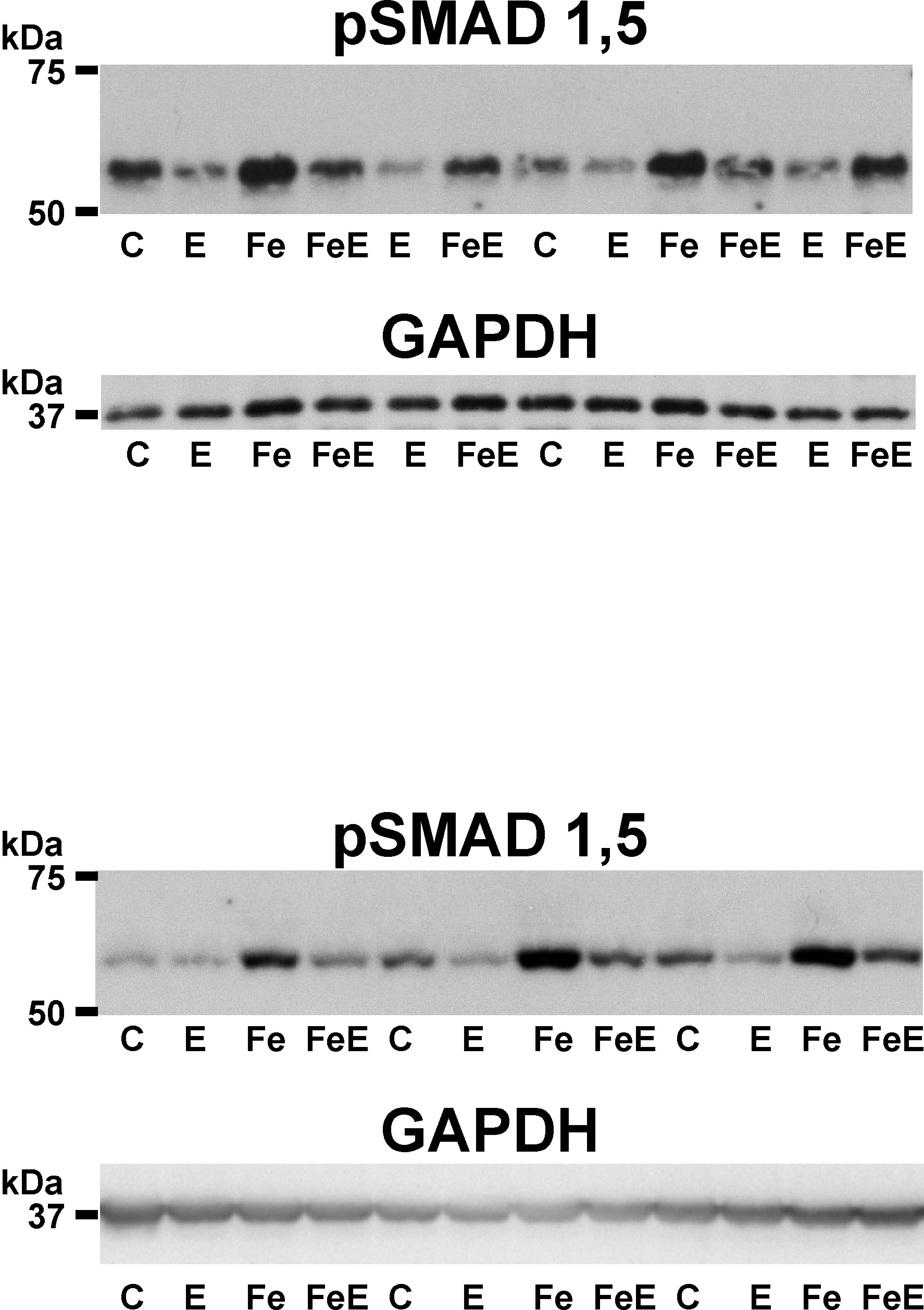


**S3 Fig.** **Additional immunoblots to Fig 1**. Additional blots demonstrating the effect of combined treatment with iron and EPO on liver pSMAD protein content. Treatment details as in Fig. 1. Column abbreviations: C: Control group, E: EPO-treated group, Fe: Iron dextran-pretreated group, FeE: Iron dextran-pretreated group administered EPO. GAPDH is used as loading control, blots were developed using AGFA CP-BU paper.
