# Supplementary material for: Effect of stimulated erythropoiesis on liver SMAD signaling pathway in iron-overloaded and iron-deficient mice
Source: PLoS One. 2019 Apr 8;14(4):e0215028. doi: 10.1371/journal.pone.0215028 (PMC6453526; doi:10.1371/journal.pone.0215028)
Supplement: S4 Fig — (DOC) [file pone.0215028.s004.doc]

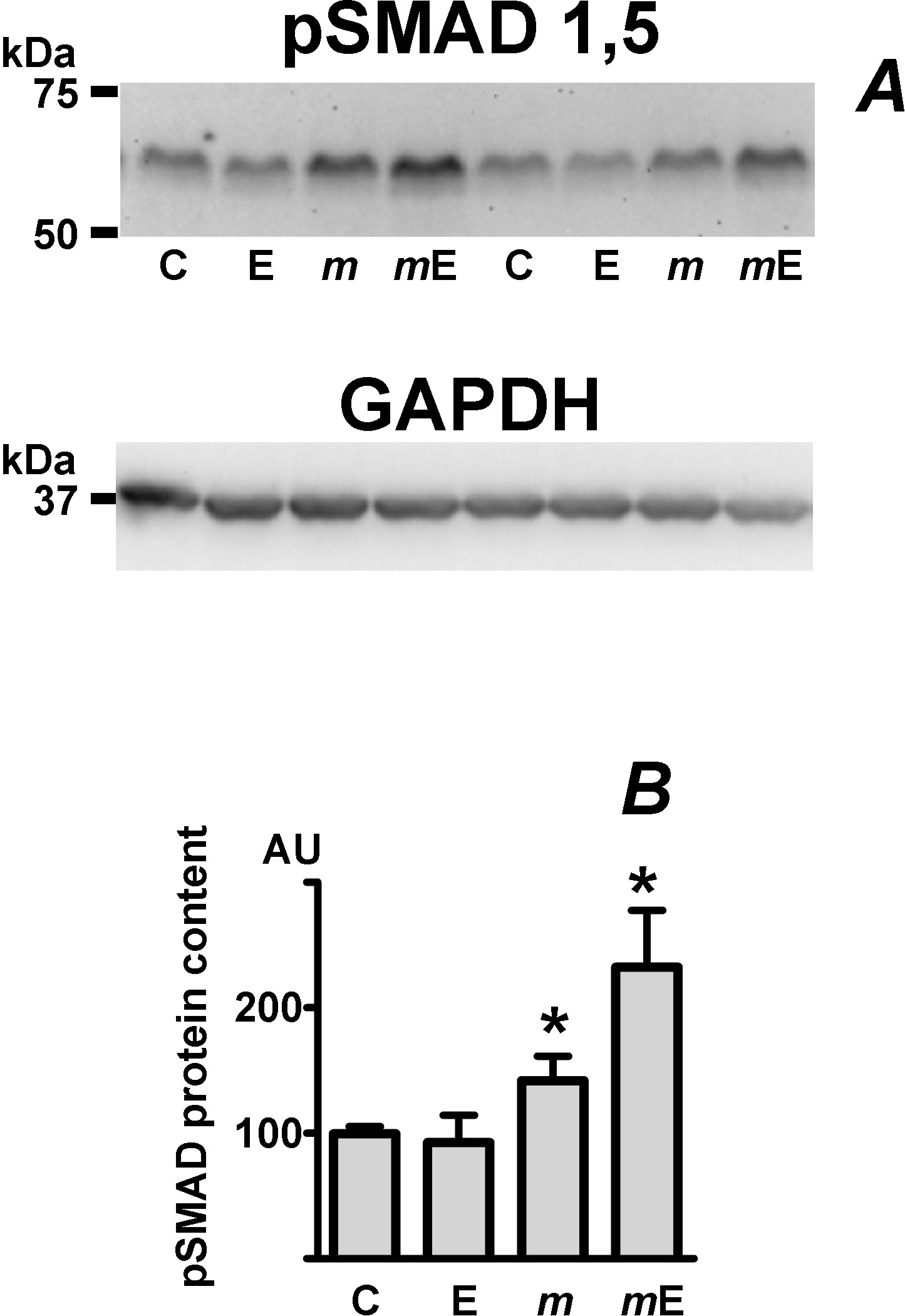


**S4 Fig. Erythropoietin does not decrease phosphorylated SMAD 1,5 protein content in *mask* mice.**

A: Immunoblot of phosphorylated SMAD 1 and 5 protein. GAPDH is used as loading control. Column abbreviations: C: Wild-type mice; E: erythropoietin-treated wild-type mice; m: *mask* mice; mE: erythropoietin-treated *mask* mice.

B: Relative content of phosphorylated SMAD protein in arbitrary units, column abbreviations as in Panel A. Asterisks denote statistically significant difference from controls, n=3.
