# Supplementary material for: Effect of stimulated erythropoiesis on liver SMAD signaling pathway in iron-overloaded and iron-deficient mice
Source: PLoS One. 2019 Apr 8;14(4):e0215028. doi: 10.1371/journal.pone.0215028 (PMC6453526; doi:10.1371/journal.pone.0215028)
Supplement: S5 Fig — (DOC) [file pone.0215028.s005.doc]

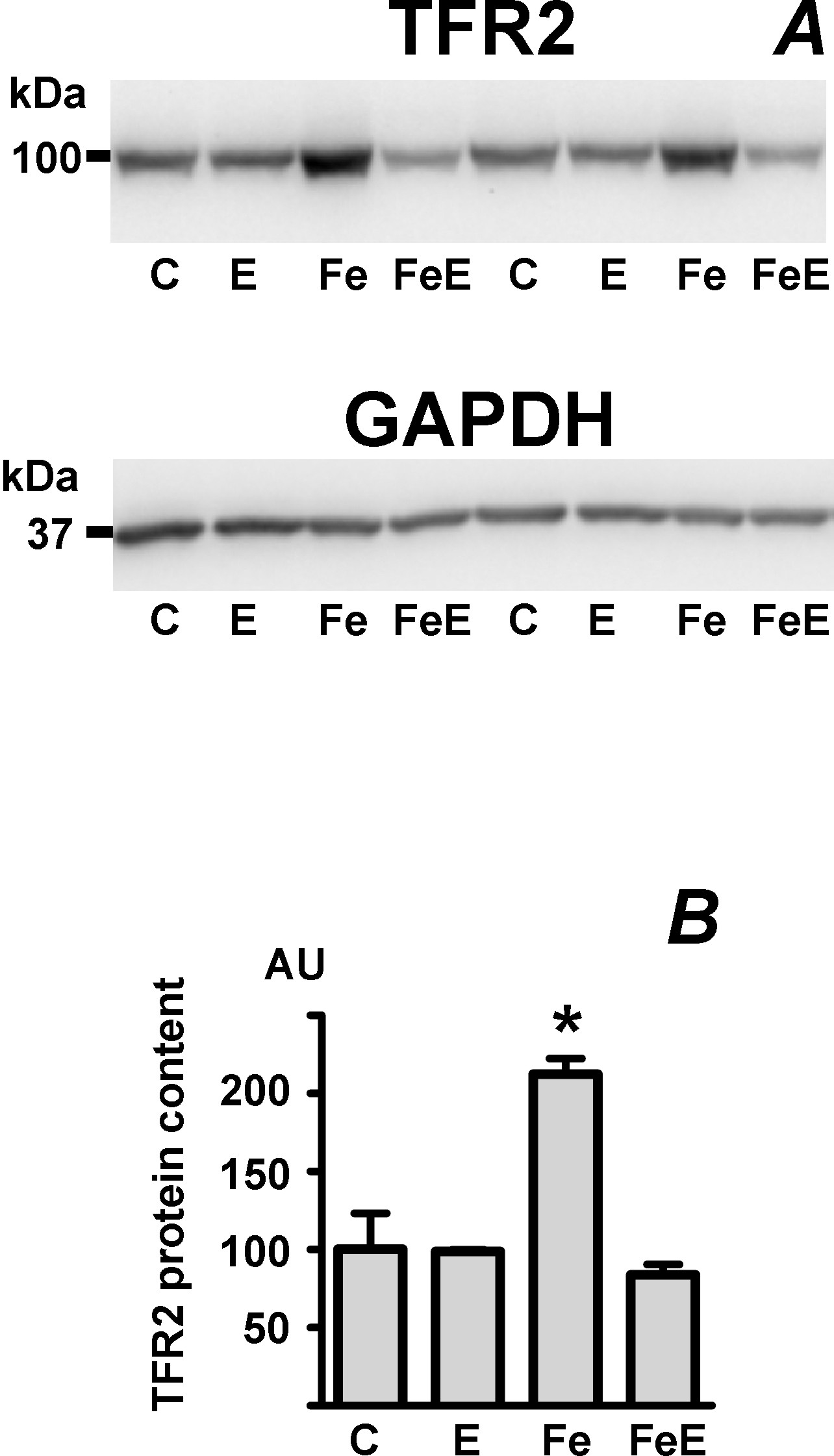


**S5 Fig. Iron treatment increases TFR2 protein content in liver microsomes.**

A: Immunoblot of TFR2 protein in liver microsomes from control mice (C), mice trated with erythropoietin (E), iron-pretreated mice (Fe) and iron-pretreated mice administered erythropoietin. Treatment details as in Materials and Methods. GAPDH is used as loading control.

B: Relative content of TFR2 protein in arbitrary units, column abbreviations as in Panel A. Asterisk denotes statistically significant difference from controls, n=3.
