# Supplementary material for: Effect of stimulated erythropoiesis on liver SMAD signaling pathway in iron-overloaded and iron-deficient mice
Source: PLoS One. 2019 Apr 8;14(4):e0215028. doi: 10.1371/journal.pone.0215028 (PMC6453526; doi:10.1371/journal.pone.0215028)
Supplement: S6 Fig — (DOC) [file pone.0215028.s006.doc]

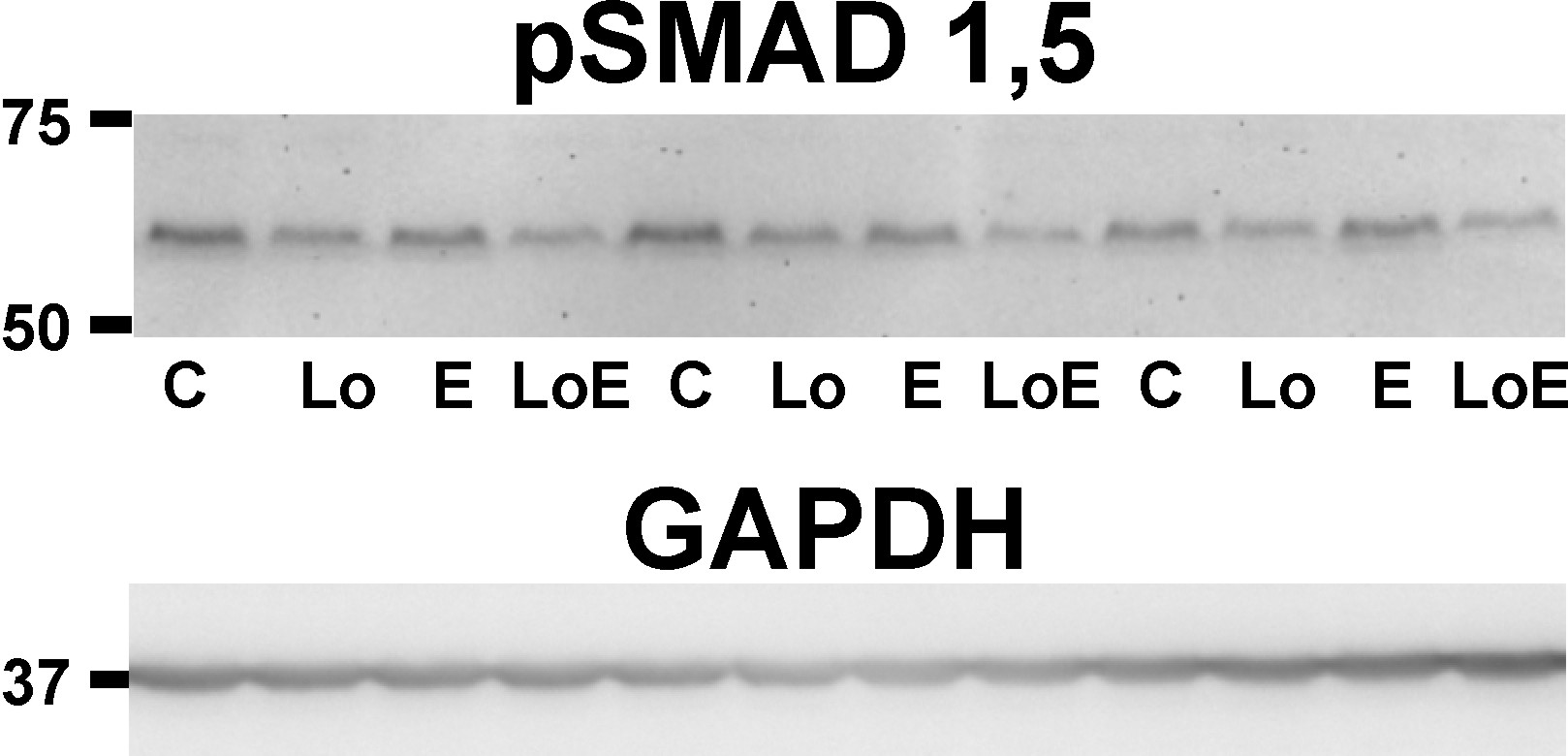


**S6 Fig.** **Additional immunoblot to Fig 5.** Additional immunoblot demonstrating the effect of combined treatment with low-iron diet and EPO on liver pSMAD protein content. Treatment details as in Fig. 5. Column abbreviations: C: Control group, Lo: Low-iron group (6 weeks on iron-deficient diet), E: EPO-treated group, LoE: Low-iron group administered EPO. GAPDH is used as loading control.
