# Supplementary material for: Effect of stimulated erythropoiesis on liver SMAD signaling pathway in iron-overloaded and iron-deficient mice
Source: PLoS One. 2019 Apr 8;14(4):e0215028. doi: 10.1371/journal.pone.0215028 (PMC6453526; doi:10.1371/journal.pone.0215028)
Supplement: S1 Table — (DOC) [file pone.0215028.s007.doc]

**S1 Table. List of primers used for PCR analysis.**

*Actb*: GACATGGAGAAGATCTGGCA and GGTCTTTACGGATGTCAACG

*Bmp6*: GAACCTGGTGGAGTACGACAA and ATGCTCCTGCAAGACTTGGTA

*Fam132b*: ATGCTGTTCGTCAAGCAGAGT and CCTTCAGCAGAACCTCAGATG

*Gdf15*: ACTGAGGTTCCTGCTGTTCCT and CCAATCTCACCTCTGGACTGA

*Hamp*: CTGAGCAGCACCACCTATCTC and TGGCTCTAGGCTATGTTTTGC

*Id1*: CGAGGTGGTACTTGGTCTGTC and CTGCAGGTCCCTGATGTAGTC

*Smad7*: GCAGGCTGTCCAGATGCTGT and GATCCCCAGGCTCCAGAAGA

*Tfr2*: CTGGGAACTGGAGACCCTTAC and AAGGAGAGCCTGAGAGGTGAC

*Twsg1*: GCTGTCACACCATGAAAACCT and GTGAAACCAGCGATACTTGGA
