# Supplementary material for: Effect of stimulated erythropoiesis on liver SMAD signaling pathway in iron-overloaded and iron-deficient mice
Source: PLoS One. 2019 Apr 8;14(4):e0215028. doi: 10.1371/journal.pone.0215028 (PMC6453526; doi:10.1371/journal.pone.0215028)
Supplement: S2 Table — (DOC) [file pone.0215028.s008.doc]

| Group | Liver iron (μg/g) | Spleen iron (μg/g) | Plasma iron (μmol/l) |
| --- | --- | --- | --- |
| Control | 91 + 27 | 318 + 186 | 21.7 + 2.5 |
| EPO | 55 + 8 | 95 + 58 * | 12.0 + 5.9 |
| Iron | 2782 + 691 * | 3850 + 631 * | 48.0 + 1.0 * |
| Iron + EPO | 2749 + 411 * | 2150 + 1299 * | 14.5 + 2.5 # |
|  |  |  |  |
| Control | 70 + 33 | 296 + 165 | 22.3 + 2.8 |
| EPO | 56 + 25 | 99 + 54 * | 10.6 + 3.8 * |
| Low | 18 + 2 * | 26 + 6 * | 30.0 + 5.0 |
| Low + EPO | 20 + 7 * | 36 + 17 * | 7.2 + 3.4 * ## |

**Supplementary Table 2.** Iron metabolism parameters in mice used in the experiments. Treatment details as in Materials and Methods. Liver and spleen iron content is expressed per wet tissue weight. Asterisks denote statistically significant difference from control group, hash tag denotes statistically significant difference from the iron-treated group; double hash tag a statistically significant difference from mice kept on an iron-deficient diet. n>3.
